# Intratype variants and high genotypic diversity of human papillomavirus with polymorphisms in the antigenic hypervariable loops of the L1 protein from women living with human immunodeficiency virus in Northeastern Brazil

**DOI:** 10.1099/jmm.0.001981

**Published:** 2025-03-19

**Authors:** Melina Vieira Alves, Guilherme Oliveira Pereira, Letícia Alves dos Santos Silva, Edilaine Dória Araújo, Brenda Evenlin Barreto da Silva, Lígia Mara Dolce de Lemos, Marcus Vinicius de Aragão Batista

**Affiliations:** 1 Laboratory of Molecular Genetics and Biotechnology (GMBio), Department of Biology, Center for Biological and Health Sciences, Federal University of Sergipe, São Cristóvão, SE, 49107-230, Brazil; 2 Graduate Program in Health Sciences, Federal University of Sergipe, Aracaju, SE, 49060-108, Brazil; 3 Department of Nursing, Center for Biological and Health Sciences, Federal University of Sergipe, Aracaju, SE, 49060-108, Brazil

**Keywords:** cervical cancer, genetic variability, human immunodeficiency virus, human papillomavirus, late major capsid protein L1, vaccine

## Abstract

**Introduction.** The human papillomavirus (HPV) is one of the main oncogenic viruses. High-risk HPV types are associated with the development of cervical cancers. In addition, it is known that some mutations in HPV genes, or variant viral lineages, have been associated with greater oncogenic risk.

**Gap statement.** The L1 protein is the major component of the viral capsid and is therefore used in currently available vaccines. However, the characterization of mutations in the L1 gene, which is relevant to increasing the knowledge of the immune escape mechanisms used by the virus, is still incipient.

**Aim.** This study aimed to characterize mutations associated with antigenic domains in the L1 protein of HPVs isolated from cervical samples of women living with HIV in Northeastern Brazil.

**Methodology.** L1 gene sequences were obtained from the samples, and the mutations and the viral variants were characterized. Phylogenetic and functional analyses of the structure of the L1 protein were carried out.

**Results.** A total of 41 HPV variant isolates were obtained, distributed among 16 different viral types. Of this, 25 non-synonymous mutations were evaluated regarding the stability of the L1 protein. It was observed that 10 of these mutations were predicted to increase, and 14 to decrease, the stability of the L1 protein and that most of them occurred in the FG hypervariable antigenic loop.

**Conclusion.** These results add useful knowledge to understanding the biological and immunological aspects of HPV variants and the impact of these mutations on the development of vaccine strategies.

## Data Availability

The data that support the findings of this study are available from the corresponding author upon reasonable request.

## Introduction

Papillomaviruses (PVs) are a group of epitheliotropic viruses with a small genome containing ~8000 bp, whose genes are divided into two groups, early expression genes (genes E) and late expression genes (genes L), as well as the upstream regulatory region. The L1 and L2 genes encode the major and minor proteins of the viral capsid, respectively [1]. Among the PVs, the human papillomavirus (HPV) is the best characterized. Currently, more than 400 viral types are known, as well as various viral intratype variants (https://pave.niaid.nih.gov/). These viruses usually cause asymptomatic, self-limiting infections that affect the skin and mucous membranes [2].

However, ~60 types of HPV show tropism for the anogenital epithelium and, of these, ~13 viral types are classified as HPVs with a high oncogenic risk [2]. Some of the lesions caused by this virus are of great clinical relevance, such as cancer of the cervix, anus, vagina, penis, oropharynx and vulva. It is estimated that 5% of cancer cases worldwide are associated with infection by HPVs with a high oncogenic risk [3]. Despite the significant contribution of this virus to the development of cancers, only a small proportion of infections develop into these diseases. It is known that factors such as the genetic variability of the virus and the integration of the viral genome, for example, are related to the maintenance of the infection and the appearance of cancers [4].

In addition, an important relationship has been observed between the genetic variability of HPV and the increased risk of developing cancers, especially in immunosuppressed individuals, as observed in those infected with the human immunodeficiency virus (HIV) [2]. In this sense, the viral capsid proteins, especially the major subunit encoded by the L1 gene, play an important role in the entry of the virus into the cell, as well as in the recognition of this micro-organism by the host immune system. Given the importance of this gene, the entire phylogeny of PVs is based on the identity of the sequences of this gene [5,6].

The L1 protein forms an arrangement of 72 subunits, capable of self-organizing to form virus-like particles (VLPs) [7]. This arrangement forms an icosahedral capsid, which is essential for the interaction between the viral surface and components of the host cell membrane [8]. Since it is a highly immunogenic protein that can be quickly identified by B lymphocytes [9], currently available vaccines use recombinant DNA technology to produce VLPs from the L1 protein [10].

Crystallographic analysis of the L1 protein shows that each unit of this protein is composed of 12 *β*-sheets, 5 *α*-helices and 6 loops [11]. These loops are exposed on the surface of this protein and are essential for the recognition and production of neutralizing antibodies by the immune system [12]. However, these loops are frequent sites of non-synonymous mutations, which form hypervariable regions in the L1 protein [13]. The presence of polymorphisms in these L1 protein loops may be related to the different affinities of the antibodies produced by the organism concerning the high diversity of aa sequences present on the viral surface [14,15]. Furthermore, the presence of these hypervariable regions may be associated with antibody-mediated immune response evasion mechanisms [16,17].

In this context, this study aimed to verify the presence of polymorphic regions in the L1 gene of different types of HPV isolated from women living with HIV in Northeastern Brazil, as well as to predict the impact of these mutations on the stability and structure of the L1 protein.

## Methods

### Collection of cervical samples

Samples were collected after the study was approved by the Research Ethics Committee of the Federal University of Sergipe, under protocol number 23374014100005545. Patients attending the Aracaju Medical Specialties Center (CEMAR), located in the state of Sergipe, Northeastern Brazil, were invited to take part in the study. Cervical samples were collected between August 2014 and October 2017. HIV-positive patients aged 18 or over were included in the study. Each patient’s endocervical secretion was collected using a cervical brush. The content obtained was used to prepare a slide for cytopathological analysis and storage in 1.5-ml tubes containing PBS buffer. In parallel, each participant answered a questionnaire about other health conditions related to HIV infection. In total, 275 women and their respective samples were included in the study. After collection, all tubes were stored in a −20 °C freezer until DNA extraction.

### DNA extraction

The Wizard® Genomic DNA Purification Kit (Promega Corporation) was used for DNA extraction according to the manufacturer’s instructions. The samples were washed using PBS buffer and centrifuged at 10 000 r.p.m. for 5 min. The pellet was suspended in 300 µl of the lysis solution, and the tubes were incubated in a water bath for 30 min at 65 °C. Then, 100 µl of protein precipitation solution was added, after which the tubes were cooled for 5 min and then centrifuged. The supernatant was then suspended and 600 µl of isopropanol was added to each tube, and the tubes were centrifuged again. The tubes received 70% ethanol and were centrifuged again. The samples were then rehydrated at 65 °C for 1 h.

After extraction, the samples were quantified using a NanoDrop™ Lite Spectrophotometer (Thermo Fisher Scientific). DNA integrity was checked by amplifying the human *β*-globin gene, using primers GH20 and PC04 (PC04-5′ CAACTTCATCCACGTTCACC 3′ and GH20-5′ GAAGAGCCAAGGACAGGTAC 3′) [18]. For this reaction, 6 µl of autoclaved ultrapure water, 1 µl of each primer (10 pmol), 10 µl of the Colourless PCR Master Mix kit (Promega Corporation) and 2 µl of DNA were used for each sample, with the steps of initial denaturation (94 °C for 5 min), denaturation (94 °C for 1 min), annealing (60 °C for 1 min), extension (72 °C for 1 min) and final extension (72 °C for 10 min) in 35 cycles.

### Molecular detection and genotyping of HPV

For the molecular identification of HPV, the primers MY09/11 [19] and the EntroA primers (EntroA F 5′ TRCCHGAYCCIAATAAGTTTG 3′ and EntroA R 5′ ACCATAGADCCACTRGGDGT 3′) were used, targeting a region of ~450 and 690 bp of two different regions of the L1 gene, respectively [20]. In the tubes of each sample, 9 µl of autoclaved ultrapure water, 1.5 µl of each primer (10 pmol), 15 µl of the PCR Master Mix kit (Promega Corporation) and 3 µl of the sample were added. The reactions involved initial denaturation (94 °C for 10 min), denaturation (94 °C for 1 min), annealing (55 °C for 1 min), extension (72 °C for 1 min) and final extension (72 °C for 10 min) in 40 cycles. Each sample was analysed in duplicate. The reaction product was visualized on a 2% agarose gel.

The PCR products from the positive samples were purified using the Wizard SV Gel and PCR Clean-Up System kit (Promega Corporation), following the manufacturer’s protocol. The purified DNA was quantified using a spectrophotometer. The samples were standardized to a concentration of 10 ng µl^−1^ and then sequenced on an ABI 3500 Genetic Analyzer sequencer (Applied Biosystems), using the BigDye Terminator Cycle Sequencing version 3.1 kit (Applied Biosystems).

The sequences were assembled using the Staden package [21], and only sequences with a Phred quality value ≥30 were used. After assembly, the sequences were aligned using the blastn tool (https://blast.ncbi.nlm.nih.gov/Blast.cgi). For each HPV type identified, the reference sequences and their respective variants were obtained from the Papillomavirus Episteme (PaVE) database (https://pave.niaid.nih.gov/) (Table S1, available in the online Supplementary Material).

### Phylogenetic and structural analysis

For the phylogenetic analysis, the sequences obtained in the study were aligned with the HPV reference sequences obtained from the PaVE and GenBank databases (https://www.ncbi.nlm.nih.gov/genbank). The alignment was carried out using mega11 software, version 11.0.9 (https://www.megasoftware.net/) [22], with the muscle alignment algorithm, maintaining the predefined default parameters. The mutations present in the sequences were identified and described.

The nt substitution model was defined using the jModelTest software version 2.1.10 (https://github.com/ddarriba/jmodeltest2/releases) [23], according to the Akaike and Bayesian information criteria. The phylogenetic tree was built using the sequences obtained experimentally and the reference sequences, using the MrBayes program (https://nbisweden.github.io/MrBayes/) [24], using the Bayesian Inference method, with the information from the model that best fit the data. In this analysis, 10 000 000 generations were used to define the posterior probabilities of the Markov chain Monte Carlo. The first 25% of the trees were discarded (burn-in) to assess the support of the branches.

The structural analysis for the non-synonymous mutations was carried out by building a three-dimensional model of the proteins for the viral types, based on homologous molecules obtained from PaVE and structures deposited in the Protein Data Bank (PDB) (https://www.rcsb.org/), using the Modeller software version 9.22 (https://salilab.org/modeller/) [25]. The stereochemical quality was checked using the Procheck software (https://www.ebi.ac.uk/thornton-srv/software/PROCHECK/). The structural and functional effect of the mutations identified in the isolates obtained was predicted using the Site Directed Mutator (SDM) server (https://veena.medschl.cam.ac.uk/sdm2). The server calculates ∆∆*G* as follows: ∆∆*G* = ∆*G*_wt_ - ∆*G*_mutant_, and negative/positive ∆∆*G* is related to unfavourable (reduced stability)/favourable (increased stability) substitutions, respectively.

## Results

### HPV detection and genotyping

During the period analysed, 275 women living with HIV attending CEMAR agreed to take part in this study. These women were not vaccinated, and they underwent a cytological examination and colposcopy and completed a questionnaire containing questions related to sociodemographic issues. The results obtained from the questionnaires and the data in the medical records are shown in Table S2.

Of all the women included in the study, 75 tested positive for HPV. Of this number, 41 isolates were sequenced, which were classified into 16 viral types distributed among 8 HPV species. In parallel with the collection of biological material, the cytopathological evaluation of the cervical samples obtained was carried out. The results of the molecular and cytopathological diagnosis are shown in Table 1.

**Table 1.** Characteristics of the cervical samples from women living with HIV in Northeastern Brazil

HPV species	HPV type	Sample ID	Accession no.	Sublineage	Cytopathology	Colposcopy	Biopsy	CD4+ count (cells/mm^3^)	HIV viral load (copies/ml)	ARV	Other infection (cytopathology)
*Alphapapillomavirus-3*	HPV 61	Sample 65	PP972052	C1	HSIL	Dense AWE	CIN I	417	Undetectable	Yes	*Gardnerella mobiluncus*
	HPV 62	Sample 12	PP972051	A1	Normal	No atypia	na	755	Undetectable	Yes	Negative
	HPV 84	Sample 337	PP972059	A1	HSIL	Dense AWE	CIN II	168	Undetectable	Yes	Negative
	HPV 86	Sample 360	PP972061	A1	Normal	No atypia	na	–	Undetectable	Yes	Negative
	HPV 89	Sample 208	PP972053	A1	Normal	No atypia	na	458	1494	Yes	Negative
	HPV 114	Sample 6	PP972055	A1	–	–	na	416	48417	Yes	Negative
	HPV 114	Sample 464	PP972054	A1	–	–	Carcinoma *in situ*	138	Undetectable	Yes	Negative
	HPV 114	Sample 443	PP972056	A1	Normal	No atypia	na	89	456174	Yes	*G. mobiluncus*
	HPV 114	Sample 48	PP972057	A1	Normal	No atypia	na	22	32918	Yes	Negative
	HPV 114	Sample 419	PP972058	A1	HSIL	AWE	na	250	Undetectable	Yes	*Trichomonas* sp.*+Gardnerella *sp.
*Alphapapillomavirus-4*	HPV 87	Sample 396	PP972060	A1	HSIL	AWE	CIN I	243	6590	Yes	Negative
*Alphapapillomavirus-5*	HPV 51	Sample 53	PP972022	A1	Normal	No atypia	na	63	235385	Yes	Negative
	HPV 51	Sample 436	PP972021	A1	Normal	AWE	na	18	72783	Yes	*G. mobiluncus*
*Alphapapillomavirus-6*	HPV 30	Sample 198	PP972047	A5	Normal	AWE	Cervicitis	1075	<40	Yes	*Candida* sp.
	HPV 30	Sample 311	PP972048	A2	–	–	na	1217	<40	No	na
	HPV 30	Sample 367	PP972050	A5	Normal	No atypia	na	–	Undetectable	Yes	Negative
	HPV 30	Sample 371	PP972049	A2	HSIL	–	CIN I	308	66039	Yes	*Trichomonas* sp.
	HPV 56	Sample 2	PP972045	B1	Normal	No atypia	na	611	<40	Yes	Negative
	HPV 56	Sample 10	PP972044	A2	Normal	No atypia	na	794	Undetectable	Yes	Negative
	HPV 56	Sample 462	PP972046	A1	Normal	No atypia	na	384	9577	Yes	Negative
*Alphapapillomavirus-9*	HPV 16	Sample 21	PP972036	A1	Normal	AWE	CIN I	776	<40	Yes	Negative
	HPV 16	Sample 38	PP972034	A1	Normal	No atypia	na	738	13126	Yes	Negative
	HPV 16	Sample 369	PP972037	A1	Normal	No atypia	na	873	288	Yes	*G. mobiluncus*
	HPV 16	Sample 412	PP972038	A1	HSIL	–	na	–	–	Yes	Negative
	HPV 16	Sample 439	PP972035	A1	Normal	Vulvar condyloma	na	214	–	No	*G. mobiluncus*
	HPV 16	Sample 458	PP972033	A1	HSIL	No atypia	na	–	Undetectable	Yes	Negative
	HPV 35	Sample 24	PP972028	A2	LSIL	No atypia	na	351	25628	Yes	Negative
	HPV 35	Sample 67	PP972031	A1	HSIL	AWE	CIN I	353	Undetectable	Yes	Negative
	HPV 35	Sample 119	PP972029	A1	HSIL	No atypia	na	392	Undetectable	Yes	Negative
	HPV 35	Sample 137	PP972032	A1	–	–	na	623	Undetectable	Yes	Negative
	HPV 35	Sample 340	PP972027	A2	LSIL	No atypia	na	254	36566	Yes	Negative
	HPV 35	Sample 447	PP972026	A2	LSIL	AWE	na	317	Undetectable	Yes	Negative
	HPV 35	Sample 456	PP972030	A1	HSIL	AWE	CIN I	714	903	Yes	*Candida* sp.
	HPV 52	Sample 386	PP972040	A1	Normal	AWE	na	221	11567	Yes	Negative
	HPV 52	Sample 393	PP972039	A1	LSIL	No atypia	na	508	344246	Yes	Negative
	HPV 52	Sample 417	PP972041	A1	Normal	No atypia	na	160	36885	No	Negative
*Alphapapillomavirus-10*	HPV 6	Sample 212	PP972043	B1	LSIL	Fine punctation	CIN I	679	47	Yes	Negative
*Alphapapillomavirus-11*	HPV 73	Sample 209	PP972042	B1	–	–	na	603	<40	Yes	na
*Alphapapillomavirus-13*	HPV 54	Sample 164	PP972023	B1	–	–	na	1348	Undetectable	Yes	na
	HPV 54	Sample 320	PP972025	C1	Normal	No atypia	na	406	Undetectable	Yes	*G. mobiluncus*
	HPV 54	Sample 465	PP972024	C1	–	No atypia	na	–	–	Yes	na

ARV, antiretroviral; AWE, acetowhite epithelium; CIN, cervical intraepithelial neoplasia; HSIL, high-grade squamous intraepithelial lesion; LSIL, low-grade squamous intraepithelial lesion; na, not available

### Phylogenetic analysis of HPV isolates

**Fig. 1.**
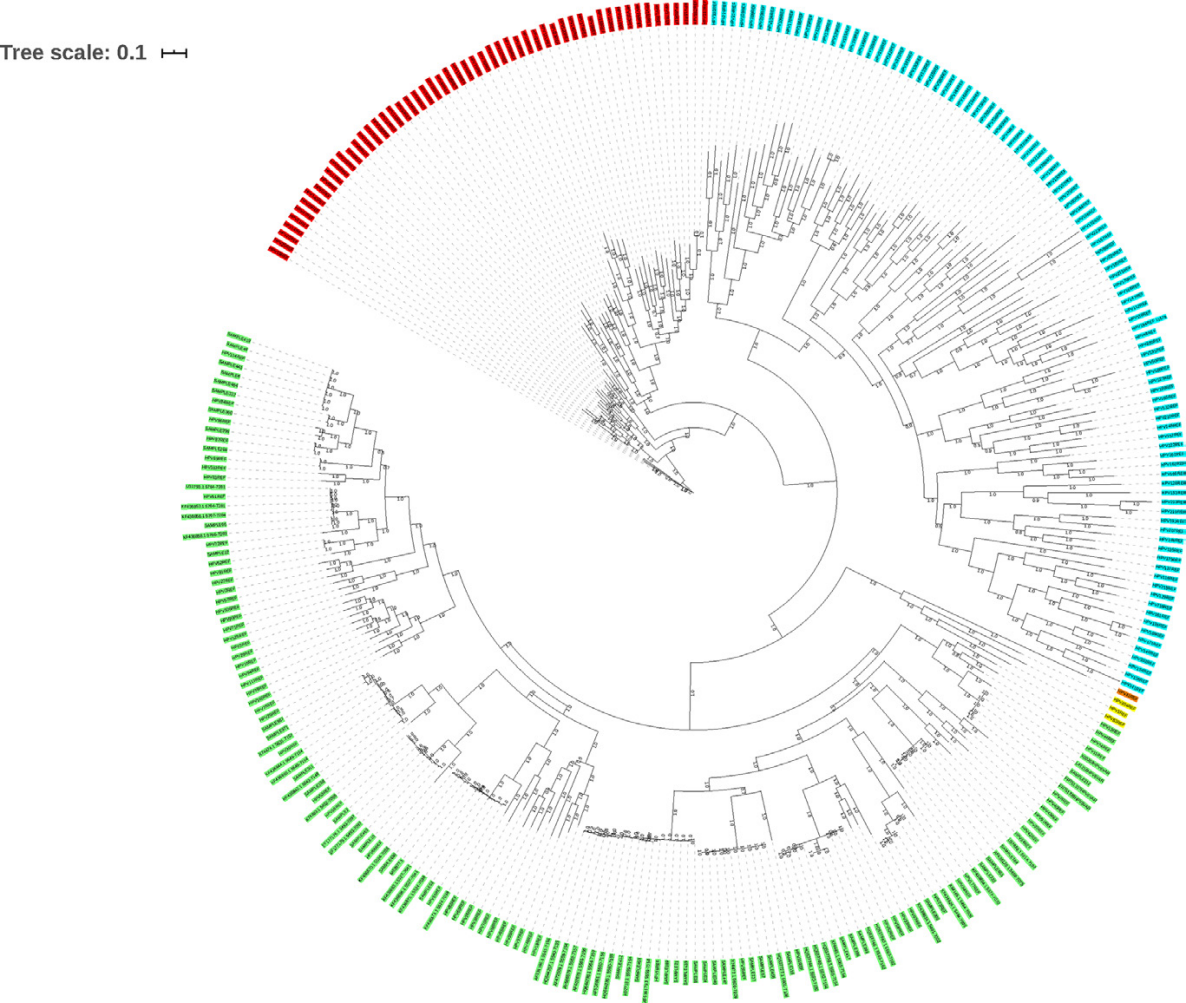
Bayesian inference phylogenetic tree based on HPV L1 gene sequences. This tree shows the clusters formed between the HPV reference sequences and the L1 gene sequences obtained from the HPV isolates of this study. Highlighted are the genera *Alphapapillomavirus* (green), *Betapapillomavirus* (red), *Gammapapillomavirus* (blue), *Mupapillomavirus* (yellow) and *Nupapillomavirus* (orange). Each node has the posterior probability values associated with the support of the branches.

The phylogenetic analysis used the 41 sequences obtained experimentally, as well as the HPV reference sequences available in the PaVE database. The most suitable substitution model for the set of sequences was GTR+I+G, which was used to build the phylogenetic tree shown in Fig. 1. All the sequences obtained in the study were grouped into their respective genera, and, in addition, the posterior probability values showed high support for most of the nodes generated.

### Annotation of L1 gene mutations

All the L1 gene sequences obtained in this study were aligned with the reference sequence of their respective viral type, obtained from the PaVE sequence database. The complete annotation of these mutations is shown in Table S3. The non-synonymous mutations were assessed concerning their impact on the hypervariable loop regions with the greatest antigenic importance for the L1 protein, identified as BC (aa 50–69), DE (aa 110–153), EF (aa 160–189), FG (aa 262–291) and HI (aa 348–360) [26]. A significant number of these mutations were located in the hypervariable antigenic loops (Table 2).

**Table 2.** Annotation of the mutations identified in the HPV isolates from this study, showing the number of mutation sites, mutation type, hypervariable antigenic loops of the L1 protein in which the mutations were found and the aa changes in the L1 protein sequence

HPV type	Sample ID	Position	Site	Mutation type	Hypervariable antigenic loop	aa change
HPV 6	Sample 212	#000	0	None	None	None
HPV 30	Sample 198	#182	2	Non-synonymous	EF (aa 160–189)	Q to P
		#280		Non-synonymous	FG (aa 262–291)	T to K
	Sample 311	#182		Non-synonymous	EF (aa 160–189)	Q to P
		#280		Non-synonymous	FG (aa 262–291)	T to K
	Sample 367	#280		Non-synonymous	FG (aa 262–291)	T to K
	Sample 371	#182		Non-synonymous	EF (aa 160–189)	Q to P
		#280		Non-synonymous	FG (aa 262–291)	T to K
HPV 35	Sample 24	#000	1	None	None	None
	Sample 67	#000		None	None	None
	Sample 119	#270		Non-synonymous	FG (aa 262–291)	T to S
	Sample 137	#000		None	None	None
	Sample 340	#000		None	None	None
	Sample 447	#000		None	None	None
	Sample 456	#000		None	None	None
HPV 51	Sample 53	#264	2	Non-synonymous	FG (aa 262–291)	V to G
		#265		Non-synonymous	FG (aa 262–291)	G to S
	Sample 436	#264		Non-synonymous	FG (aa 262–291)	V to G
HPV 52	Sample 386	#000	0	None	None	None
	Sample 393	#000		None	None	None
	Sample 417	#000		None	None	None
HPV 54	Sample 164	#137		Non-synonymous	DE (aa 110–153)	A to T
	Sample 320	#189	4	Non-synonymous	EF (aa 160–189)	S to T
		#270		Non-synonymous	FG (aa 262–291)	D to E
		#280		Non-synonymous	FG (aa 262–291)	L to P
	Sample 465	#189		Non-synonymous	EF (aa 160–189)	S to T
		#270		Non-synonymous	FG (aa 262–291)	D to E
		#280		Non-synonymous	FG (aa 262–291)	L to P
HPV 56	Sample 2	#000	0	None	None	None
	Sample 10	#000		None	None	None
	Sample 462	#000		None	None	None
HPV 61	Sample 65	#90	6	Non-synonymous	BC (aa 50–90)	G to S
		#177		Non-synonymous	EF (aa 160–189)	A to T
		#267		Non-synonymous	FG (aa 262–291)	V to T
		#271		Non-synonymous	FG (aa 262–291)	A to T
		#275		Non-synonymous	FG (aa 262–291)	S to T
		#286		Non-synonymous	FG (aa 262–291)	A to T
HPV 73	Sample 209	#272	1	Non-synonymous	FG (aa 262–291)	D to E
HPV 84	Sample 337	#183	1	Non-synonymous	EF (aa 160–189)	Y to S
HPV 86	Sample 360	#122	6	Non-synonymous	DE (aa 110–153)	A to I
		#177		Non-synonymous	EF (aa 160–189)	T to A
		#180		Non-synonymous	EF (aa 160–189)	S to A
		#188		Non-synonymous	EF (aa 160–189)	D to E
		#264		Non-synonymous	FG (aa 262–291)	F to Y
		#277		Non-synonymous	FG (aa 262–291)	E to D
HPV 87	Sample 396	#179	1	Non-synonymous	EF (aa 160–189)	N to T
HPV 89	Sample 208	#141	1	Non-synonymous	EF (aa 160–189)	V to A
HPV 114	Sample 6	#000	0	None	None	None
	Sample 48	#000		None	None	None
	Sample 419	#000		None	None	None
	Sample 443	#000		None	None	None
	Sample 464	#000		None	None	None

IUPAC code: Y (tyrosine); V (valine); A (alanine); N (asparagine); P (proline), F (phenylalanine); E (glutamic acid); S (serine); Q (glutamine); G (glycine); I (isoleucine); L (leucine); K (lysine); T (threonine), D (aspartate).

For the samples of HPV 6, 52, 56 and 114, no non-synonymous mutations were identified in the hypervariable loops of the L1 protein. For some viral types, only one isolate was obtained, in which only one non-synonymous mutation was identified: HPV 73 (sample 209) substitution D272E (FG loop), HPV 84 (sample 337) substitution Y183S (EF loop), HPV 73 (sample 209) substitution D272E (FG loop), HPV 87 (sample 396) substitution N179T (EF loop) and HPV 89 (sample 208) substitution V141A (EF loop).

All the samples of HPV 30 (samples 198, 311, 367 and 371) showed a change of the aa threonine to lysine at position 280, included in the FG loop region. In addition to this mutation, samples 198, 311 and 371 show the same change from glutamine to proline at position 182 (EF loop). For the samples of HPV 35, only sample 119 had a non-synonymous mutation at position 270 (FG loop), in which a threonine aa was changed to serine.

The two samples of HPV 51 (samples 53 and 436) had non-synonymous mutations in the FG region, at position 264, with the aa valine being changed to glycine. In addition to this mutation, sample 53 had the G265S change in the same region (FG). Two samples of HPV 54 (samples 320 and 465) had the same aa substitutions: S189T (EF loop), D270E (FG loop) and L280P (FG loop). Only sample 164 presented the A137T substitution (DE loop).

**Fig. 2.**
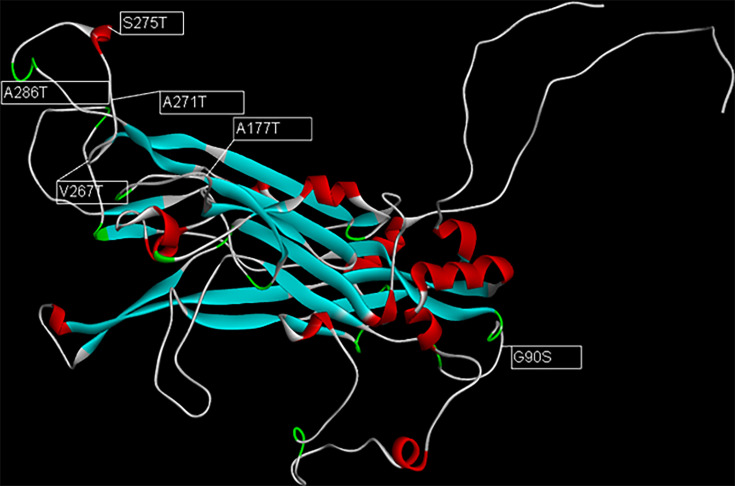
Three-dimensional structure of the HPV 61 L1 protein highlighting the aa substitutions identified in sample 65.

The only sample identified for HPV 61 (sample 65) presented the highest number of non-synonymous mutations, most of which were in the FG region. The substitutions observed were G90S (BC loop), A177T (EF loop), V267T (FG region), A271T (FG region), S275T (FG region) and A286T (FG region) (Fig. 2). A high number of non-synonymous mutations were also observed in HPV 86 (sample 360), in which most of the mutations described occurred in the EF loop region. The substitutions identified were A122I (DE loop), T177A (EF loop), S180A (EF loop), D188E (EF loop), F264Y (FG loop) and E277F (FG loop).

### Structural analysis of the L1 protein

Based on the non-synonymous mutations identified, a structural analysis of the stability of the L1 protein was carried out using homology-based molecular modelling. Only for HPV 35 was used a structure template obtained from the PDB database (ID: 2R5J). The stereochemical quality of the models was evaluated using the Ramachandran plot, which showed that all models presented satisfactory quality (Table S4). All L1 protein 3D structure models are shown in Figs S1–S10.

Fig. 2 shows the structure of the HPV 61 L1 protein, one of the viral types with the highest number of mutations (indicated by arrows in the image, with their respective substitutions). These mutations were predicted to reduce the stability of the protein, most of which occurred in the FG hypervariable loop. Of the 25 non-synonymous mutations identified, 10 were predicted to increase the stability of the L1 protein. The substitution that promoted the greatest stability of the protein, as measured by the ΔΔ*G* value (1.5), was the A122I substitution in HPV 86. On the other hand, the substitution with the lowest ΔΔ*G* value (−3.64) was the G90S exchange in HPV 61 (Table 3).

**Table 3.** Conformational thermodynamic stability analysis of the HPV L1 protein variants identified in this study

HPV type	Mutation	Ramachandran value (%)	ΔΔ*G*	Potential stability change
HPV 30	Q182P	95.19	0.8	Increased stability
	T280K		0	Neutral change
HPV 35	T270S	95.11	0.07	Increased stability
HPV 51	V264G	95.14	−0.72	Reduced stability
	G265S		−0.47	Reduced stability
HPV 54	A137T	92.96	−0.11	Reduced stability
	S189T		0.8	Increased stability
	D270E		1.49	Increased stability
	L280P		−1.46	Reduced stability
HPV 61	G90S	91.29	−3.64	Reduced stability
	A177T		−0.09	Reduced stability
	V267T		−0.85	Reduced stability
	A271T		−0.61	Reduced stability
	S275T		−0.14	Reduced stability
	A286T		−0.83	Reduced stability
HPV 73	D272E	94.01	1.49	Increased stability
HPV 84	Y183S	90.22	−0.4	Reduced stability
HPV 86	A122I	91.53	1.5	Increased stability
	T177A		0.86	Increased stability
	S180A		0.4	Increased stability
	D188E		−0.35	Reduced stability
	F264Y		0.5	Increased stability
	E277D		−0.39	Reduced stability
HPV 87	N179T	91.47	0.04	Increased stability
HPV 89	V141A	91.20	−1.16	Reduced stability

## Discussion

In this study, it was possible to identify the HPV types in a cohort of women living with HIV in Northeastern Brazil. In addition, it was possible to molecularly characterize the L1 protein of these isolates, allowing us to advance our knowledge of the aspects involved in the viral infection process and the relationship between the viral variants and the patterns of cervical alterations in these patients. This relationship is quite relevant to the population analysed, as several studies indicate that women living with HIV have a higher risk of acquiring the infection and a lower chance of HPV clearance [27,28] as well as a higher risk of developing cervical neoplasms [29], especially those with low CD4+ T lymphocyte counts [30]. Some studies have indicated that the opposite relationship is also true, as it has been observed that women with HPV infection have a higher risk of HIV infection, possibly due to the local inflammatory response and involvement of the cervical mucosa [31,33].

The mechanisms involved in the increased risk of cervical neoplasms in women living with HIV compared to HIV-negative women are not yet fully understood. Some hypotheses suggest that this increase may be associated with chromosomal instabilities [34] and immunosuppression generated by HIV [35]. In parallel, *in vitro* studies suggest that HIV-1 proteins may act in the modulation of HPV gene expression and modifications in the gene expression of infected cells [36,37].

Furthermore, epidemiological studies of the association between HPV-HIV co-infection show that HIV-positive individuals have a higher risk of infection with high-risk oncogenic HPV (HR-HPV) [38,39], which does not necessarily include HPV types 16 and 18, the most prevalent HR-HPV in cases of cervical neoplasia in the general population [40]. In this study, the most commonly detected high-risk viral types in our sample set were HPV 35 (*n*=7) and HPV 16 (*n*=6). Other HR-HPV types detected were HPV 56, HPV 52 and HPV 51, also identified in population samples of women living with HIV in other regions of the world [41].

It is known that HPV adhesion and entry into the keratinocytes located in the basal layer of the epithelium and the stem cells present in this tissue is mediated by the interaction between the viral capsid proteins, L1 and L2. The proteins involved in this process include integrins *α*6 and *β*4, CD63 and CD151 proteins, annexin A2 and epidermal and keratinocyte growth factor receptors [42]. In addition, viral capsid proteins are the main targets for vaccine development due to the high immunogenic capacity of these molecules [43]. Given the importance of these genes, it is expected that the genes encoding L1 and L2 proteins will suffer great Darwinian selective pressure when compared to other HPV genes [44].

The main sites where mutations are found in the L1 gene are regions called hypervariable loops, small aa sequences that form secondary structures that project onto the surface of L1 and correspond to important regions of neutralizing epitopes [14]. Mutations in these loops interfere with the stability of the protein and also with the immune response, especially the antibody-mediated response [12,45]. In the present study, the position of the mutations in the aa sequence was evaluated to understand whether there was a relationship between these hypervariable loops and clinical outcomes.

Of the women who tested positive for HPV 16, two had high-grade cervical lesions and three had alterations on colposcopy examination. As for the women infected with HPV 35, all seven samples had intraepithelial lesions and colposcopy alterations. A single woman infected with HPV 52 (*n*=3) had a low-grade lesion and the two cases of HPV 51 had normal cytology, while the only case of HPV 61 had a high-grade lesion and CIN2/3 in biopsy. Our results also showed that certain low-risk HPV types were associated with cervical lesions, which suggested that in people living with HIV, low-risk HPV infections should not be disregarded, as they may contribute to a greater oncogenic risk than in the general population.

In Brazil, although recommended, the HPV vaccine for women living with HIV is not mandatory. Currently available HPV vaccines provide coverage against four or nine HPV types, which include the types with the highest oncogenic risk and the most prevalent. In our study, we detected unusual viral types (when we consider the prevalence in the general population, in Brazil and in the world), which are not covered by commercially available vaccines. In addition, L1 variants of HPV 30, 35, 51, 54, 61, 73, 84, 86, 87 and 89 presented mutations, and they are not included in the current HPV vaccines. It is possible that there is a protective effect of the vaccine against uncommon viral types; however, other studies are needed to evaluate the protective effect of immunization against HPV for women living with HIV against viral types other than those included in the vaccines.

As for the functional and structural analysis of HR-HPVs, no non-synonymous mutations were found in any of the HPV 16 samples. In another study, in which similar analyses were carried out involving the L1 and L2 genes of HPV 16, a low occurrence of mutations was observed in these genes, of which only a portion was of the non-synonymous type. It is possible that due to the great selective pressure acting on these genes, as well as the fact that HPVs use enzymes from the host cell to carry out their replication, an enzymatic machinery that is less prone to errors, these genes are more conserved [46].

The non-synonymous substitution identified in one of the HPV 35 samples (T270S, loop FG) was previously described in a molecular analysis study of cervical samples from HIV-positive and HIV-negative women in Canada in 2010 [47]. The conservation of this mutation might be associated with an increase in the stability of the L1 protein, but this hypothesis needs to be further assessed.

Two HPV 51 samples had non-synonymous mutations, all located in the FG loop region, and both mutations resulted in reduced stability of the L1 protein. Other non-synonymous mutations in the L1 gene of HPV 51 were described for other sites in a study of isolates obtained in China. In this analysis, the H119Y and N176S substitutions affected hypothetical B lymphocyte epitopes [48]. To the best of our knowledge, no other study of the genetic variability of the L1 gene of HPV 51 was performed. For the other high-risk HPV types (HPV 52 and HPV 56), no non-synonymous mutations were observed.

In this study, a high diversity of HPV types was observed, as well as a significant number of positive samples for low-risk HPV types, and in some of these cases, these infections were associated with high-grade lesions. Other studies have also shown a high prevalence of low-risk HPV types in high-grade lesions [49,50].

In two women (samples 164 and 465) with HPV 54 infection (*n*=3), high-grade cervical lesions were observed. For these isolates, it was not possible to observe a direct relationship between the mutations that generate an increase in the stability of the L1 protein and the neoplastic potential, since sample 164 presented only one non-synonymous mutation in the DE loop, associated with a reduction in stability, while sample 465 presented three mutations in the EF and FG loops, two of which promoted an increase in stability and one caused a reduction in the stability of the L1 protein. No other studies were found describing the genetic variability of L1 in HPV 54, possibly because it is an HPV with a low oncogenic risk and is therefore less frequently identified in cervical lesions in the general population.

An ambiguous relationship was also observed in sample 337 (HPV 84), in which, even though there was a reduction in the stability of L1, the woman carrying this infection had a high-grade cervical lesion, while in sample 360 (HPV 86), although most of the non-synonymous mutations made the L1 protein more stable, there was no clinical association with a higher-grade lesion. When analysing the samples with probable oncogenic risk (HPV 30 and HPV 73), all the mutations identified in samples 311 and 371 (HPV 30) and sample 209 (HPV 73) favoured the stability of the L1 protein, and, in these cases, the patients presented cytology compatible with high-grade alterations.

As for the hypervariable loops involved, it was found a high prevalence of non-synonymous mutations in the FG (19) and EF (12) loops (*n*=34). It has been suggested that the DE and FG loop regions of HPV 16 are related to interaction with certain regions of the L2 protein and binding to heparin sulphate proteoglycans, which is essential for the virus to enter cells, while the EF loop appears to be associated with binding between L1 protein units for capsomer formation [51].

Although the results of this study contribute to an understanding of the genetic diversity of HPV and its possible relationship with the development of high-grade lesions and cervical cancer, this study has certain limitations. As this was a cross-sectional study, it was not possible to determine the onset of HPV infections and the appearance of cytological alterations, as well as the evolution of the immune status of the patients included in the study. Furthermore, due to the diagnostic method applied, it was not possible to detect more than one viral type present in the sample.

## Conclusions

This study allowed us to identify the most prevalent HPV types in the cohort of women living with HIV in the state of Sergipe, Northeastern Brazil, as well as provide insights into the association between the genetic variability of the L1 protein of the viral types identified in these samples and the clinical outcome presented by the patients.

Despite the limitations presented by this study, an important relationship was observed between the low-risk and probable HR-HPV types in terms of the development of high-grade intraepithelial lesions, demonstrating the need not to neglect HPV infections in women co-infected with HIV.

Finally, the detection of mutations in the hypervariable loop regions and their impact on the biology of this virus also reflect the importance of developing new studies associated with the impact of these mutations on vaccine strategies.

## supplementary material

10.1099/jmm.0.001981 Uncited Supplementary Material 1.

## Abbreviations

HIV: human immunodeficiency virus
HPV: human papillomavirus
HR-HPV: high-risk oncogenic HPV
PaVE: Papillomavirus Episteme
PDB: Protein Data Bank
PVs: papillomaviruses
VLPs: virus-like particles

## References

[1] Willemsen A, Bravo IG (2019). Origin and evolution of papillomavirus (onco)genes and genomes. Philos Trans R Soc Lond B Biol Sci.

[2] Dube Mandishora RS, Gjøtterud KS, Lagström S, Stray-Pedersen B, Duri K (2018). Intra-host sequence variability in human papillomavirus. Papillomavirus Res.

[3] Szymonowicz KA, Chen J (2020). Biological and clinical aspects of HPV-related cancers. Cancer Biol Med.

[4] Lagström S, Umu SU, Lepistö M, Ellonen P, Meisal R (2019). TaME-seq: an efficient sequencing approach for characterisation of HPV genomic variability and chromosomal integration. Sci Rep.

[5] de Villiers E-M, Fauquet C, Broker TR, Bernard H-U, zur Hausen H (2004). Classification of papillomaviruses. Virology.

[6] Van Doorslaer K, Tan Q, Xirasagar S, Bandaru S, Gopalan V (2013). The papillomavirus episteme: a central resource for papillomavirus sequence data and analysis. Nucleic Acids Res.

[7] Kirnbauer R, Booy F, Cheng N, Lowy DR, Schiller JT (1992). Papillomavirus L1 major capsid protein self-assembles into virus-like particles that are highly immunogenic. Proc Natl Acad Sci USA.

[8] Roos N, Breiner B, Preuss L, Lilie H, Hipp K (2020). Optimized production strategy of the major capsid protein HPV 16L1 non-assembly variant in *E. coli*. *Protein Expr Purif*.

[9] Yazdani Z, Rafiei A, Valadan R, Ashrafi H, Pasandi M (2020). Designing A potent L1 protein-based HPV peptide vaccine: a bioinformatics approach. Comput Biol Chem.

[10] de Oliveira CM, Fregnani JHTG, Villa LL (2019). HPV Vaccine: updates and highlights. Acta Cytol.

[11] Oumeslakht L, Ababou M, Badaoui B, Qmichou Z (2021). Worldwide genetic variations in high-risk human papillomaviruses capsid L1 gene and their impact on vaccine efficiency. Gene.

[12] Bishop B, Dasgupta J, Klein M, Garcea RL, Christensen ND (2007). Crystal structures of four types of human papillomavirus L1 capsid proteins: understanding the specificity of neutralizing monoclonal antibodies. J Biol Chem.

[13] Wang A, Li N, Zhou J, Chen Y, Jiang M (2018). Mapping the B cell epitopes within the major capsid protein L1 of human papillomavirus type 16. Int J Biol Macromol.

[14] Chen XS, Garcea RL, Goldberg I, Casini G, Harrison SC (2000). Structure of small virus-like particles assembled from the L1 protein of human papillomavirus 16. Mol Cell.

[15] Varsani A, Williamson AL, Jaffer MA, Rybicki EP (2006). A deletion and point mutation study of the human papillomavirus type 16 major capsid gene. Virus Res.

[16] Da Silva DM, Pastrana DV, Schiller JT, Kast WM (2001). Effect of preexisting neutralizing antibodies on the anti-tumor immune response induced by chimeric human papillomavirus virus-like particle vaccines. Virology.

[17] El Aliani A, El Abid H, Kassal Y, Khyatti M, Attaleb M (2020). HPV16 L1 diversity and its potential impact on the vaccination-induced immunity. Gene.

[18] Bell DA, Taylor JA, Paulson DF, Robertson CN, Mohler JL (1993). Genetic risk and carcinogen exposure: a common inherited defect of the carcinogen-metabolism gene glutathione S-transferase M1 (GSTM1) that increases susceptibility to bladder cancer. J Natl Cancer Inst.

[19] Castle PE, Schiffman M, Gravitt PE, Kendall H, Fishman S (2002). Comparisons of HPV DNA detection by MY09/11 PCR methods. J Med Virol.

[20] Barros GS, Araujo ED, Santos FLSG, Batista MVA (2020). Application of an entropy-based computational strategy to identify genomic markers for molecular detection and typing of human papillomavirus. *Infect Genet Evol*.

[21] Dear S, Staden R (1991). A sequence assembly and editing program for efficient management of large projects. Nucleic Acids Res.

[22] Tamura K, Stecher G, Kumar S (2021). MEGA11: molecular evolutionary genetics analysis version 11. Mol Biol Evol.

[23] Darriba D, Taboada GL, Doallo R, Posada D (2012). jModelTest 2: more models, new heuristics and parallel computing. Nat Methods.

[24] Ronquist F, Teslenko M, van der Mark P, Ayres DL, Darling A (2012). MrBayes 3.2: efficient bayesian phylogenetic inference and model choice across a large model space. Syst Biol.

[25] Eswar N, Webb B, Marti-Renom MA, Madhusudhan MS, Eramian D (2006). Comparative protein structure modeling using modeller. Curr Protoc Bioinform.

[26] Shen-Gunther J, Cai H, Zhang H, Wang Y (2019). Abundance of HPV L1 intra-genotype variants with capsid epitopic modifications found within low- and high-grade pap smears with potential implications for vaccinology. Front Genet.

[27] Ahdieh L, Klein RS, Burk R, Cu-Uvin S, Schuman P (2001). Prevalence, incidence, and type-specific persistence of human papillomavirus in human immunodeficiency virus (HIV)-positive and HIV-negative women. J Infect Dis.

[28] Liu G, Sharma M, Tan N, Barnabas RV (2018). HIV-positive women have higher risk of human papilloma virus infection, precancerous lesions, and cervical cancer. AIDS.

[29] Stelzle D, Tanaka LF, Lee KK, Ibrahim Khalil A, Baussano I (2021). Estimates of the global burden of cervical cancer associated with HIV. Lancet Glob Health.

[30] Hessol NA, Holly EA, Efird JT, Minkoff H, Weber KM (2013). Concomitant anal and cervical human papillomavirusV infections and intraepithelial neoplasia in HIV-infected and uninfected women. AIDS.

[31] Houlihan CF, Larke NL, Watson-Jones D, Smith-McCune KK, Shiboski S (2012). Human papillomavirus infection and increased risk of HIV acquisition. a systematic review and meta-analysis. AIDS.

[32] Looker KJ, Rönn MM, Brock PM, Brisson M, Drolet M (2018). Evidence of synergistic relationships between HIV and human papillomavirus (HPV): systematic reviews and meta-analyses of longitudinal studies of HPV acquisition and clearance by HIV status, and of HIV acquisition by HPV status. J Int AIDS Soc.

[33] Zayats R, Murooka TT, McKinnon LR (2022). HPV and the risk of HIV acquisition in women. Front Cell Infect Microbiol.

[34] Wistuba II, Syed S, Behrens C, Duong M, Milchgrub S (1999). Comparison of molecular changes in cervical intraepithelial neoplasia in HIV-positive and HIV-indeterminate subjects. Gynecol Oncol.

[35] de Sanjosé S, Brotons M, Pavón MA (2018). The natural history of human papillomavirus infection. Best Pract Res Clin Obstet Gynaecol.

[36] Vernon SD, Hart CE, Reeves WC, Icenogle JP (1993). The HIV-1 tat protein enhances E2-dependent human papillomavirus 16 transcription. Virus Res.

[37] Lien K, Mayer W, Herrera R, Padilla NT, Cai X (2022). HIV-1 Proteins gp120 and tat promote epithelial-mesenchymal transition and invasiveness of HPV-Positive and HPV-negative neoplastic genital and oral epithelial cells. Microbiol Spectr.

[38] Camargo M, Del Río‐Ospina L, Soto‐De León SC, Sánchez R, Pineda‐Peña AC (2018). association of HIV status with infection by multiple HPV types. *Tropical Med Int Health*.

[39] Monteiro JC, Fonseca RR de S, Ferreira TC de S, Rodrigues LLS, da Silva ARB (2021). prevalence of high risk HPV in HIV-infected women from belém, Pará, amazon region of Brazil: a cross-sectional study. Front Public Health.

[40] McKenzie ND, Kobetz EN, Hnatyszyn J, Twiggs LB, Lucci JA (2010). Women with HIV are more commonly infected with non-16 and -18 high-risk HPV types. Gynecol Oncol.

[41] Clifford GM, Tully S, Franceschi S (2017). Carcinogenicity of human papillomavirus (HPV) types in HIV-positive Women: a meta-analysis From HPV infection to cervical cancer. *Clin Infect Dis*.

[42] Aksoy P, Gottschalk EY, Meneses PI (2017). HPV entry into cells. Mutat Res Rev Mutat Res.

[43] Yousefi Z, Aria H, Ghaedrahmati F, Bakhtiari T, Azizi M (2021). An update on human papilloma virus vaccines: history, types, protection, and efficacy. Front Immunol.

[44] Van Doorslaer K (2013). Evolution of the papillomaviridae. Virology.

[45] Kamuyu G, Coelho da Silva F, Tenet V, Schussler J, Godi A (2024). Global evaluation of lineage-specific human papillomavirus capsid antigenicity using antibodies elicited by natural infection. Nat Commun.

[46] Yue Y, Yang H, Wu K, Yang L, Chen J (2013). Genetic variability in L1 and L2 genes of HPV-16 and HPV-58 in Southwest China. PLoS One.

[47] Cornut G, Gagnon S, Hankins C, Money D, Pourreaux K (2010). Polymorphism of the capsid L1 gene of human papillomavirus types 31, 33, and 35. J Med Virol.

[48] Ye M, Li S, Luo P, Tang X, Gong Q (2022). Genetic variation of E6, E7, and L1 genes of human papillomavirus 51 from central China. J Med Virol.

[49] de Mattos AT, de Freitas LB, Lima BMC, Miranda AE, Spano LC (2011). Diversity and uncommon HPV types in HIV seropositive and seronegative women attending an STI clinic. *Braz J Microbiol*.

[50] Entiauspe LG, Seixas FK, Nunes EM, Rodrigues FM, Dellagostin OA (2014). Uncommon non-oncogenic HPV genotypes, TP53 and MDM2 genes polymorphisms in HIV-infected women in Southern Brazil. *Braz J Infect Dis*.

[51] Bissett SL, Godi A, Beddows S (2016). The DE and FG loops of the HPV major capsid protein contribute to the epitopes of vaccine-induced cross-neutralising antibodies. Sci Rep.

