# Supplementary material for: Intratype variants and high genotypic diversity of human papillomavirus with polymorphisms in the antigenic hypervariable loops of the L1 protein from women living with human immunodeficiency virus in Northeastern Brazil
Source: J Med Microbiol. 2025 Mar 19;74(3):001981. doi: 10.1099/jmm.0.001981 (PMC11923093; doi:10.1099/jmm.0.001981)
Supplement: Uncited Supplementary Material 1. [file jmm-74-01981-s001.pdf]

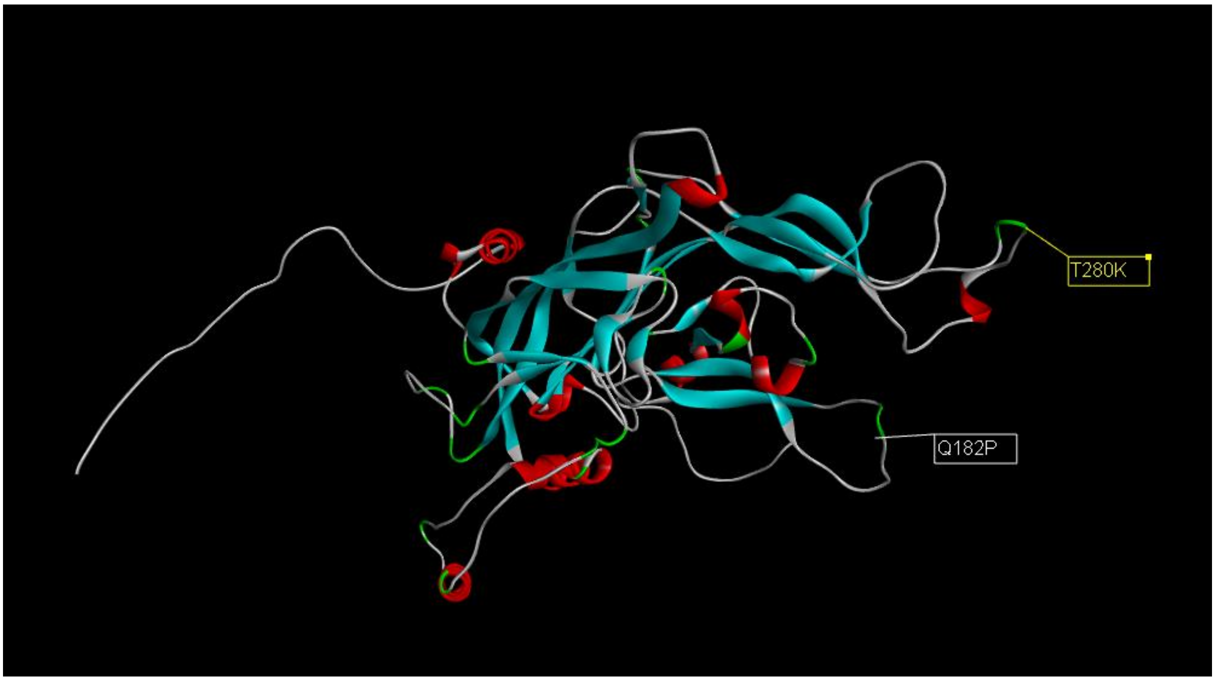

Supplementary Figure S1. Modeled HPV 30 protein and location of mutations.

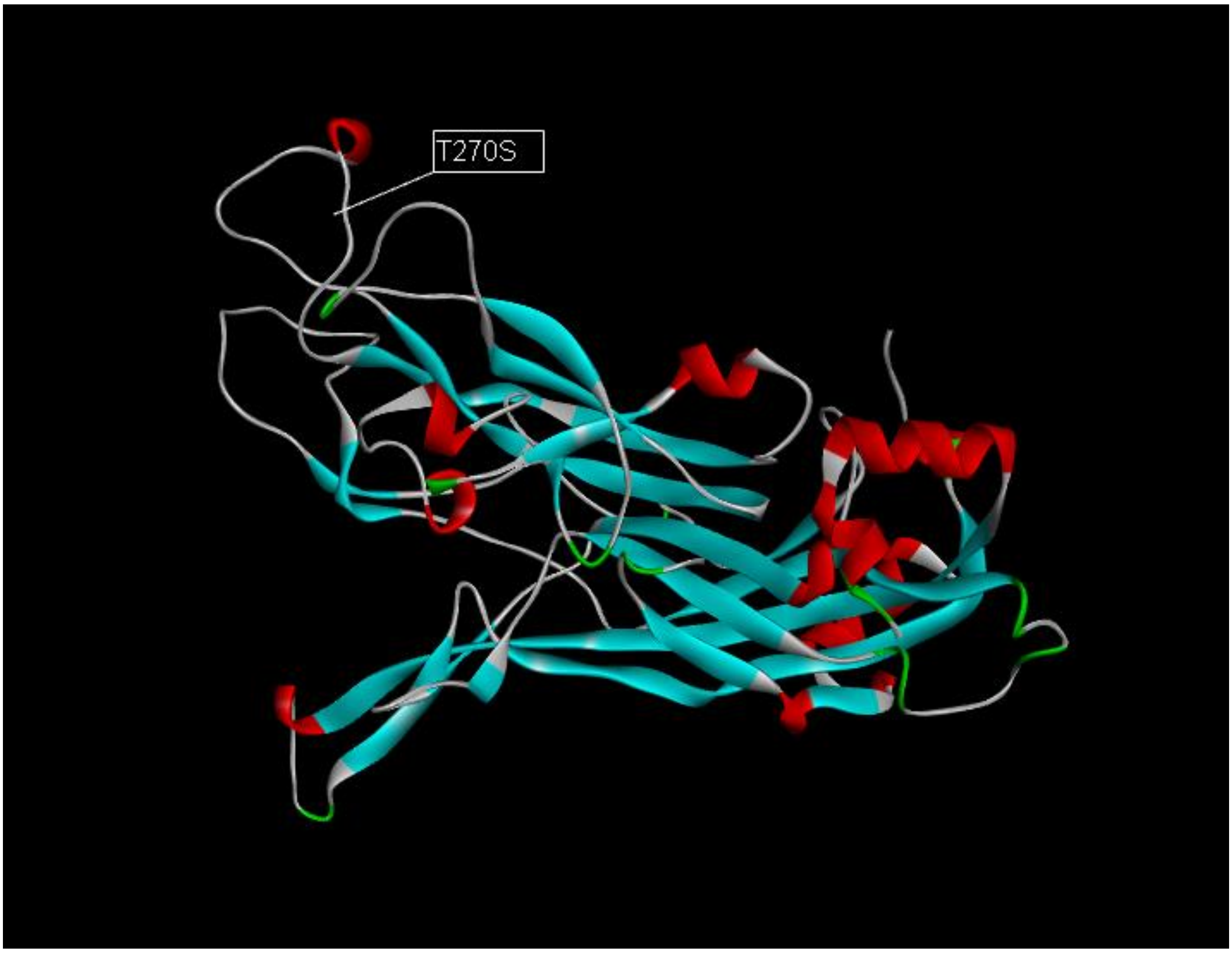

Supplementary Figure S2. Modeled HPV 35 protein and location of mutations.

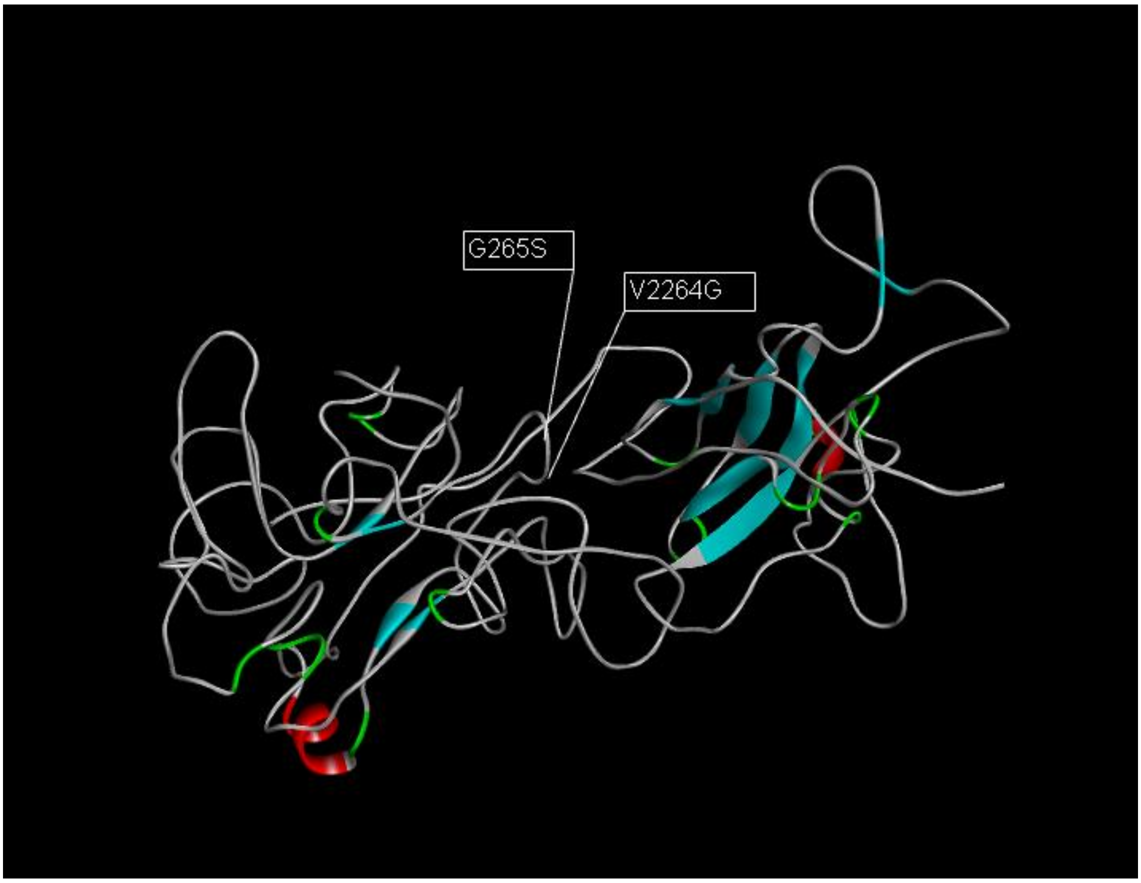

Supplementary Figure S3. Modeled HPV 51 protein and location of mutations.

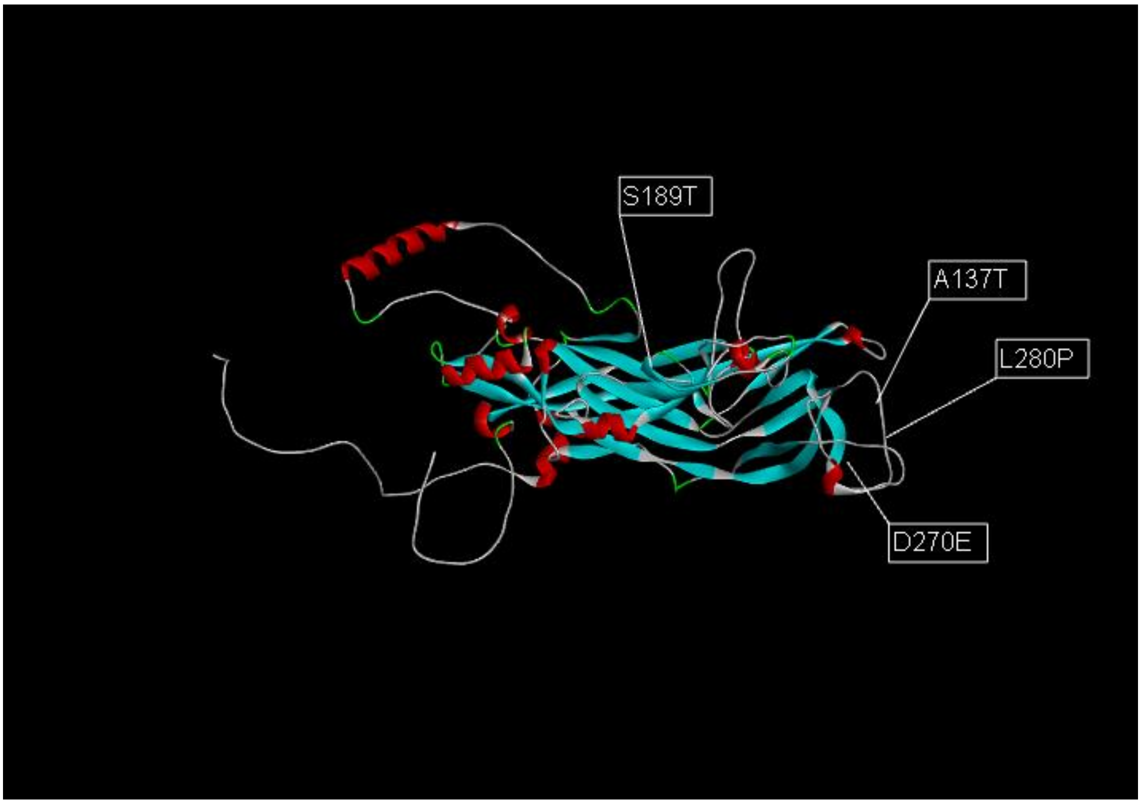

Supplementary Figure S4. Modeled HPV 54 protein and location of mutations.

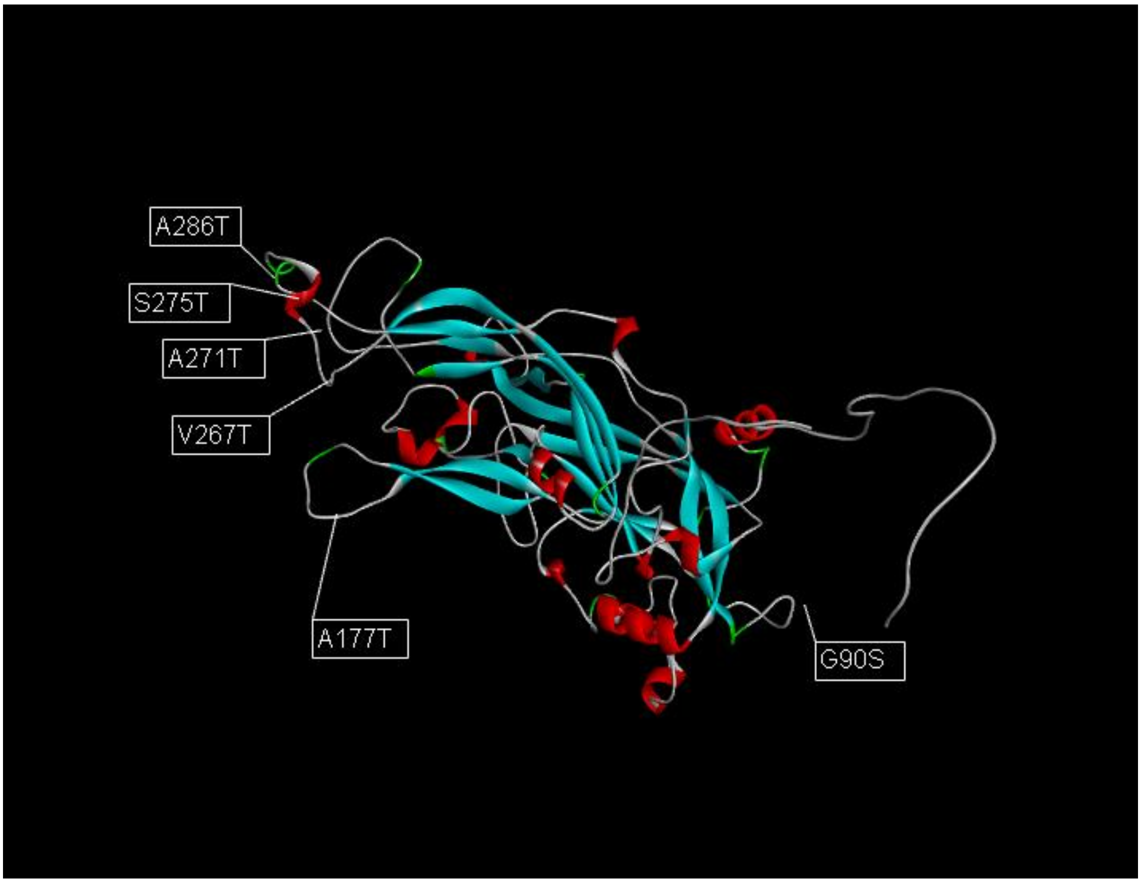

Supplementary Figure S5. Modeled HPV 61 protein and location of mutations.

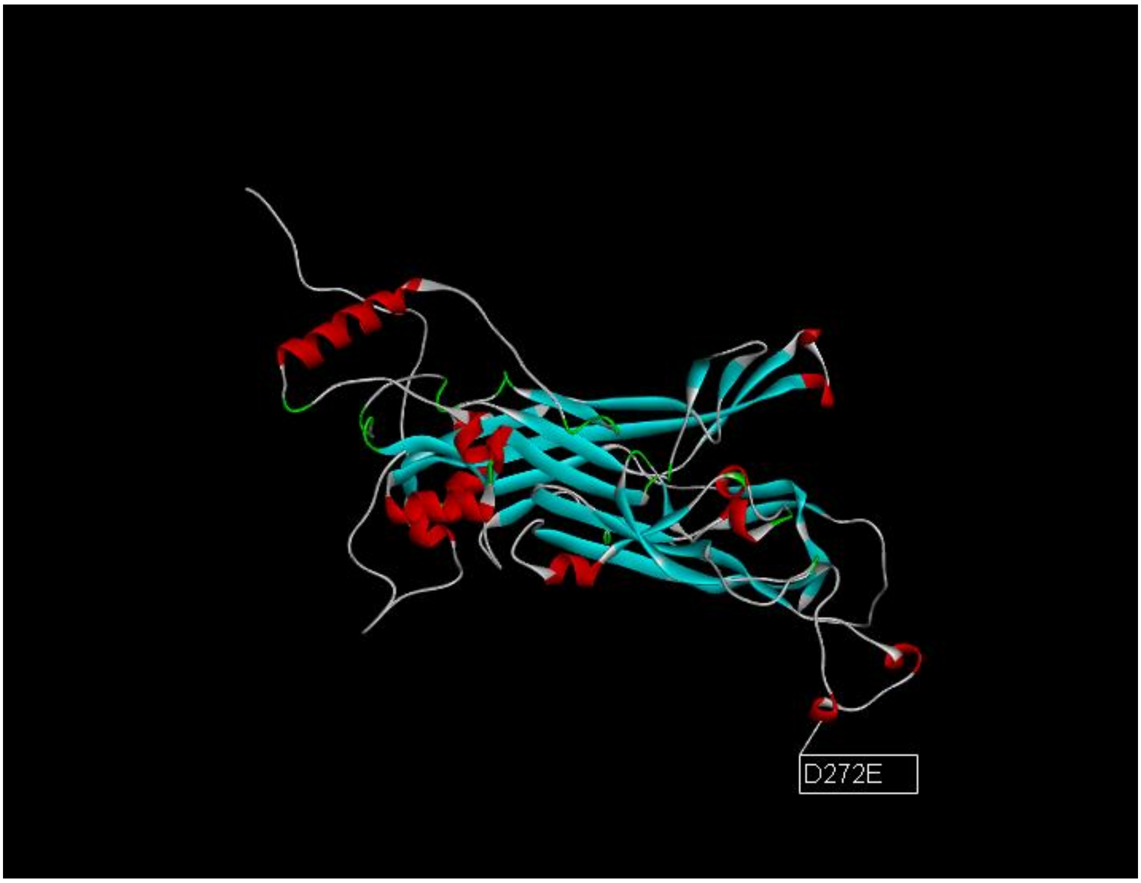

Supplementary Figure S6. Modeled HPV 73 protein and location of mutations.

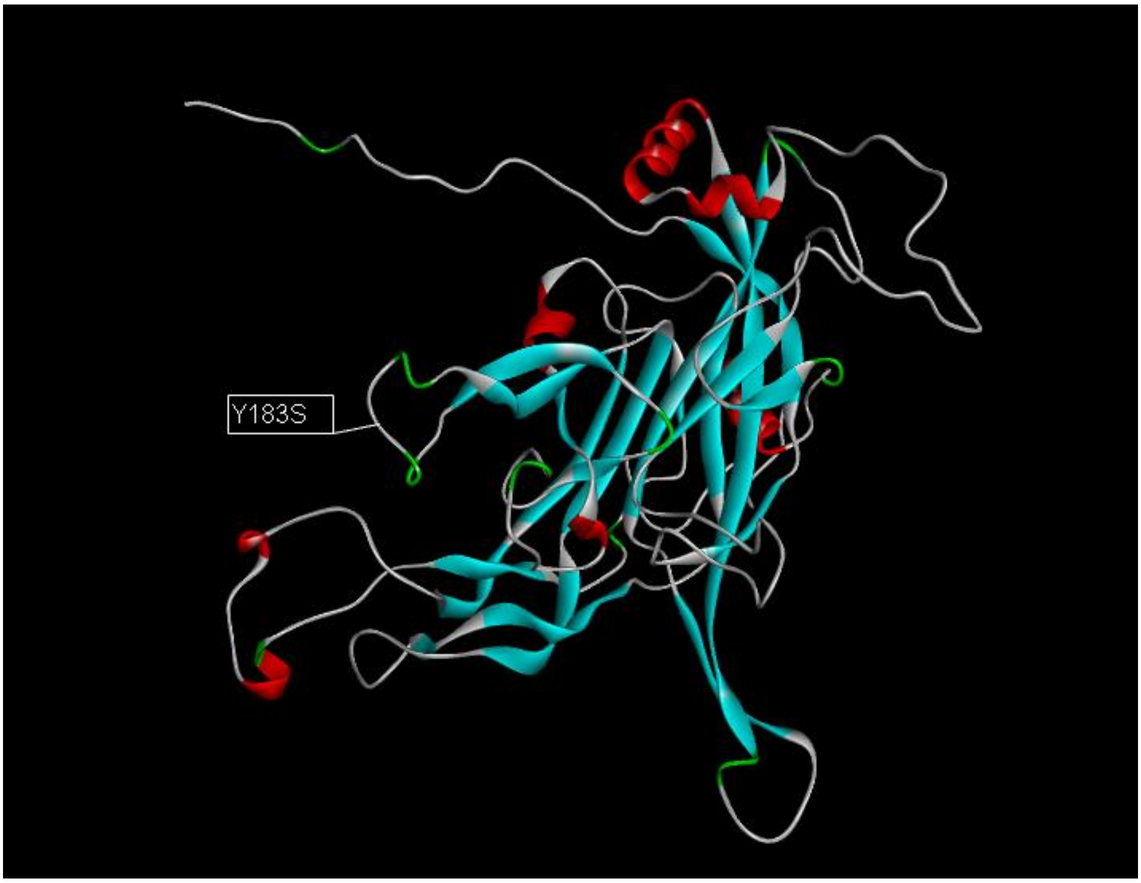

Supplementary Figure S7. Modeled HPV 84 protein and location of mutations.

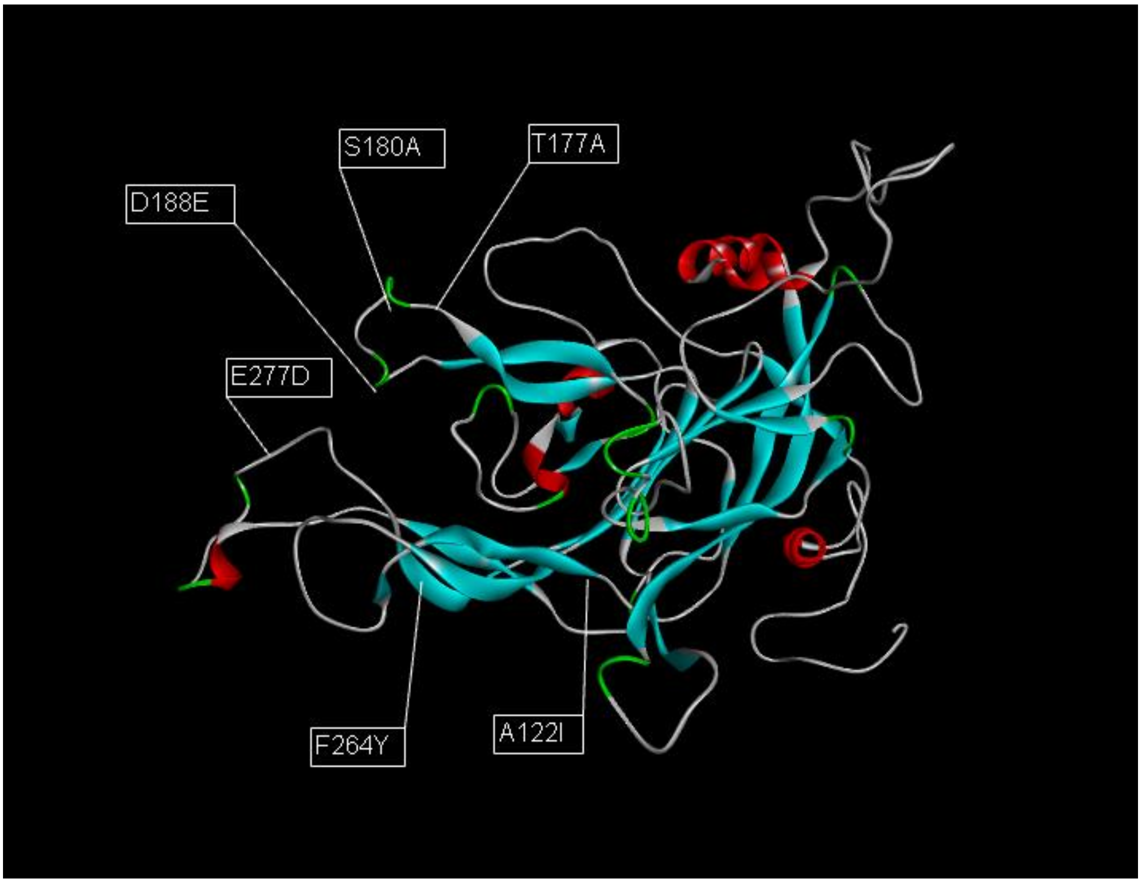

Supplementary Figure S8. Modeled HPV 86 protein and location of mutations.

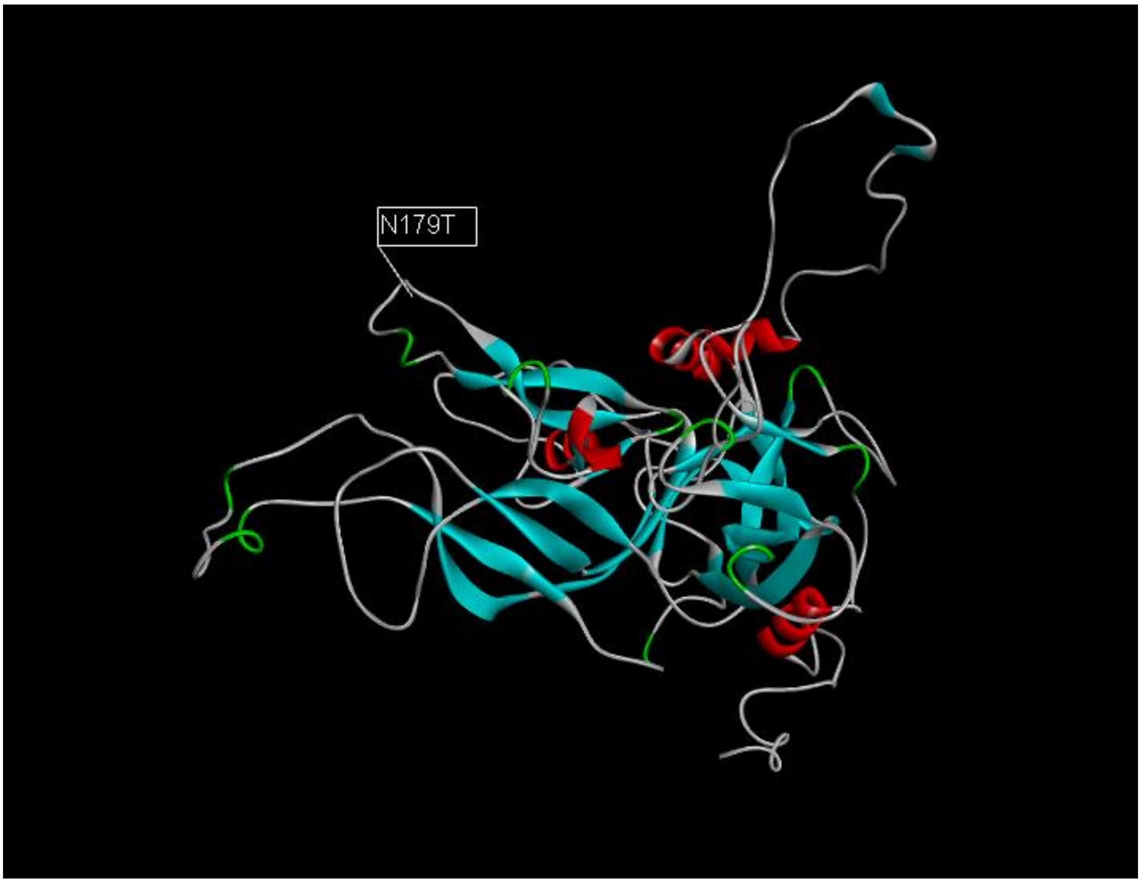

Supplementary Figure S9. Modeled HPV 87 protein and location of mutations.

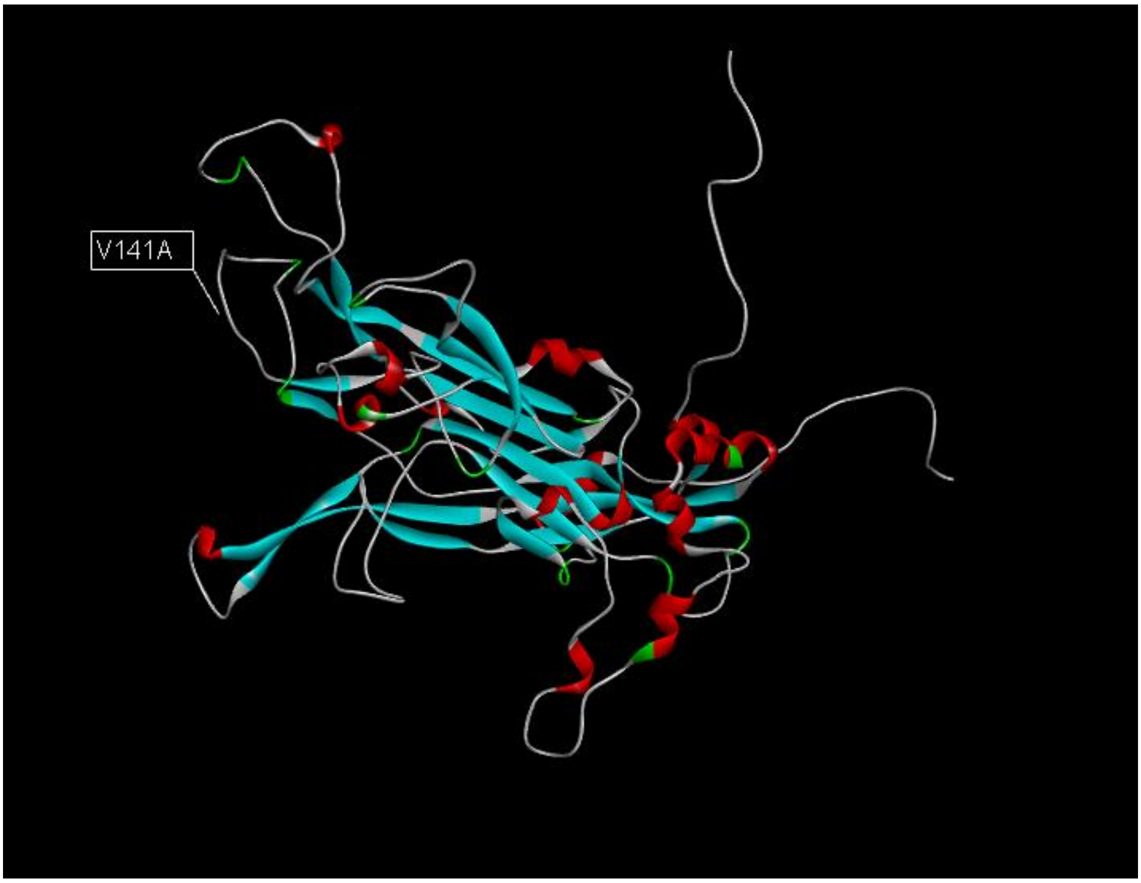

Supplementary Figure S10. Modeled HPV 89 protein and location of mutations.

Supplementary Table S1. Classification of HPV types identified after local alignment using the BLASTn tool.

	NCBI BLASTn					
**Species**	**Samples**	**types of HPV**	**Query Cover**	**E-value**	**Per. Ident.**	**Access**
Alphapapillomavirus-3	SAMPLE 65	HPV 61	100%	0.0	99.84%	KF436857.1
	SAMPLE 12	HPV 62	100%	0.0	99.35%	KU298924.1
	SAMPLE 337	HPV 84	100%	0.0	96.69%	AF293960.1
	SAMPLE 360	HPV 86	100%	0.0	94.29%	AF349909.1
	SAMPLE 208	HPV 89	100%	0.0	99.67%	KU298945.1
	SAMPLE 6	HPV 114	100%	0.0	99.83%	GQ244463.1
	SAMPLE 48	HPV 114	100%	0.0	100.00%	GQ244463.1
	SAMPLE 419	HPV 114	100%	0.0	100.00%	GQ244463.1
	SAMPLE 443	HPV 114	100%	0.0	100.00%	GQ244463.1
	SAMPLE 464	HPV 114	100%	0.0	99.44%	GQ244463.1
Alphapapillomavirus-4	SAMPLE 396	HPV87	100%	0.0	99.68%	KU298942.1
Alphapapillomavirus-5	SAMPLE 53	HPV 51	100%	0.0	100.00%	MH577961.1
	SAMPLE 436	HPV 51	100%	0.0	99.60%	MH577961.1
Alphapapillomavirus-6	SAMPLE 198	HPV 30	100%	0.0	99.33%	KF436841.1
	SAMPLE 311	HPV 30	99%	0.0	99.84%	KF436843.1
	SAMPLE 367	HPV 30	100%	0.0	99.69%	KF436841.1
	SAMPLE 371	HPV 30	99%	0.0	99.84%	KF436843.1
	SAMPLE 2	HPV 56	1	0.0	99.65%	KX645780.1
	SAMPLE 10	HPV 56	100%	2,00E-53	98.36%	KX645772.1
	SAMPLE 462	HPV 56	99%	0.0	99.84%	KX514418.1
Alphapapillomavirus-9	SAMPLE 21	HPV 16	100%	0.0	100.00%	LC456627.1
	SAMPLE 38	HPV 16	100%	0.0	100.00%	LC456627.1
	SAMPLE 369	HPV 16	100%	0.0	100.00%	LC456627.1
	SAMPLE 412	HPV 16	100%	0.0	100.00%	LC456627.1
	SAMPLE 439	HPV 16	100%	0.0	100.00%	LC456627.1
	SAMPLE 458	HPV 16	100%	0.0	99.83%	LC456627.1
	SAMPLE 24	HPV 35	100%	0.0	100.00%	KX514416.1
	SAMPLE 67	HPV 35	100%	0.0	100.00%	JN104062.1
	SAMPLE 119	HPV 35	100%	0.0	100.00%	GQ479033.1
	SAMPLE 137	HPV 35	100%	0.0	100.00%	JN104062.1
	SAMPLE 340	HPV 35	100%	0.0	100.00%	KX514416.1
	SAMPLE 447	HPV 35	99%	0.0	100.00%	KX514416.1
	SAMPLE 456	HPV 35	99%	0.0	99.84%	HQ537724.1
	SAMPLE 386	HPV 52	100%	0.0	100.00%	LC270063.1

	SAMPLE 393	HPV 52	100%	0.0	99.84%	LC270063.1
	SAMPLE 417	HPV 52	100%	0.0	99.84%	LC270063.1
Alphapapillomavirus-10	SAMPLE 212	HPV 6	100%	0.0	100.00%	KX514423.1
Alphapapillomavirus-11	SAMPLE 209	HPV 73	100%	8,00E-177	98.85%	KF436833.1
Alphapapillomavirus-13	SAMPLE 164	HPV 54	100%	4,00E-134	100.00%	KF436893.1
	SAMPLE 320	HPV 55	100%	0.0	100.00%	KU298914.1
	SAMPLE 465	HPV 56	100%	0.0	99.84%	KU298914.1

Characteristics	Percentage (%)
**Age**	
<20 years old	5.6
20 to 40 years old	57.28
>40 years old	37.12
**Cytology**	
Normal	73.48
LSIL	3.03
HSIL	6.44
ASC-H	1.14
ASCUS	1.52
No result	15.91
**Colposcopy**	
No atypia	70.83
AWE	11.36
Dense AWE	0.76
Vulvar condyloma	3.03
Vaginal condyloma	0.38
Atypical blood vessels	0.38
Fine punctation	0.38
No result	12.88
**Cervical biopsy**	
Normal	0.76
CIN 1	5.32
CIN 2	0.76
CIN 3	0.38
Carcinoma in situ	1.52
Condyloma	0.38
Squamous Metaplasia	0.76
Chronic cervicitis	2.28
No result	87.83

Supplementary Table S3: Annotation of L1 gene mutations.

HPV30

	Sample ID	Variable sites									
	(position)	240	429	534	545	558	639	666	839	867	882
nt.	HPV30REF	C	T	G	A	T	A	A	C	A	T
	SAMPLE198	-	.	.	C	.	.	G	A	-	-
	SAMPLE311	G	.	A	C	G	G	G	A	.	C
	SAMPLE367	-	C	.	.	.	.	G	A	.	-
	SAMPLE371	G	.	A	C	G	G	G	A	.	-
aa.	(position)	80	143	178	182	186	213	333	280	289	294
	HPV30REF	P	S	A	Q	P	L	P	T	S	T
	SAMPLE198	.	.	.	P	.	.	.	K	.	.
	SAMPLE311	.	.	.	P	.	.	.	K	.	.
	SAMPLE367	.	.	.	.	.	.	.	K	.	.
	SAMPLE371	.	.	.	P	.	.	.	K	.	.

HPV35

	Sample ID	Variable sites						
	(position)	294	339	765	816	817	879	885
nt.	HPV35REF	G	G	G	A	A	T	T
	SAMPLE137	.	A	A	.	.	.	-
	SAMPLE67	.	A	A	.	.	-	-
	SAMPLE24	A	A	A	.	.	.	-
	SAMPLE447	A	A	A	.	.	.	C
	SAMPLE340	A	A	A	.	.	.	-
	SAMPLE119	.	A	A	.	T	.	-
	SAMPLE456	.	A	A	G	.	C	C
aa.	(position)	98	113	255	269	270	293	295
	HPV35REF	L	L	R	E	T	Y	T
	SAMPLE137	.	.	.	.	.	.	.
	SAMPLE67	.	.	.	.	.	.	.
	SAMPLE24	.	.	.	.	.	.	.
	SAMPLE447	.	.	.	.	.	.	.
	SAMPLE340	.	.	.	.	.	.	.
	SAMPLE119	.	.	.	.	S	.	.
	SAMPLE456	.	.	.	.	.	.	.

HPV54

	Sample ID (position)	Variable sites													
		243	244	283	285	318	330	366	387	409	414	420	441	492	504
nt.	HPV54REF	A	C	T	A	G	G	A	A	G	C	G	T	C	C
	SAMPLE164	T	T	C	T	A	A	G	C	A	T	A	C	T	-
	SAMPLE320	T	T	C	T	A	A	G	C	.	T	A	C	T	.
	SAMPLE465	T	T	C	T	A	A	G	C	.	T	A	C	T	A
aa.	(position)	81	82	95		106	110	122	129	137	138	140	147	164	168
	HPV54REF	G	L	L		R	L	K	A	A	D	R	Y	H	G
	SAMPLE164	.	.	.		.	.	.	.	T	.	.	.	.	-

SAMPLE320	.	.	.	.	.	.	.	.	.	.	.	.	.
SAMPLE465	.	.	.	.	.	.	.	.	.	.	.	.	.

Continuation

537	565	591	621	622	625	636	642	658	660	735	750	774	810	839
T	T	A	A	A	C	A	T	G	A	C	A	T	C	T
-	-	-	-	-	-	-	-	-	-	-	-	-	-	-
A	A	G	T	G	T	T	C	A	T	T	G	C	G	C
A	A	G	T	G	T	T	C	A	T	T	G	C	G	C
179	189	197	207	208	209	211	214	220		245	250	258	270	280
G	S	V	K	T	L	S	S	V	V	Y	Q	N	D	L
-	-	-	-	-	-	-	-	-	-	-	-	-	-	-
.	T	.	N	A	.	.	.	I		.	.	.	E	P
.	T	.	N	A	.	.	.	I		.	.	.	E	P

HPV61

	Sample ID (position)	Variable sites 268	357	369	444	462	525	529	576	625	685	687	693
nt.	HPV61REF	G	T	G	G	G	T	G	A	A	A	A	G
	SAMPLE65	A	C	.	T	A	.	A	C	.	G	.	A
aa.	(position)	90	119	123	148	154	175	177	192	209	229		231
	HPV61REF	G	T	P	V	Q	A	A	T	I	I		K
	SAMPLE65	S	.	.	.	.	.	T	.	.	V		.

Continuation

732	756	762	799	800	807	811	823	825	830	855	856
A	C	A	G	T	T	G	T	T	A	G	G
G	A	G	A	C	.	A	A	C	.	A	A
244	252	254	267		269	271	275		277	285	286
G	R	E	V		G	A	S		Y	A	A
.	.	.	T		.	T	T		.	.	T

HPV73

	Sample ID (position)	Variable sites 654	678	729	741	766	816	864
nt.	HPV73REF	A	C	C	T	C	C	T
	SAMPLE209	G	T	T	C	A	A	G
aa.	(position)	218	226	243	247	256	272	288
	HPV73REF	V	T	S	Y	R	D	V
	SAMPLE209	.	.	.	.	.	E	.

HPV86

	Sample ID (position)	Variable sites 300	303	336	360	364	365	366	378	414	435	468
nt.	HPV86REF	T	G	A	A	G	C	C	G	C	A	G
	SAMPLE360	G	A	T	C	A	T	T	A	T	G	A
	(position)	100	101	112	120		122		126	138	145	156

aa.										
	HPV86REF	T	E	V	V	A	P	S	V	K
	SAMPLE360	.	.	.	.	I	.	.	.	.

Continuation

492	510	528	529	531	538	564	649	657	678	688	694	696
C	T	C	A	A	T	C	C	A	A	G	G	C
A	A	T	G	T	G	G	T	C	G	A	A	T
164	170	176	177		180	188	217	219	226	230	232	
G	G	G	T		S	D	L	E	L	A	V	
.	.	.	A		A	E	.	D	.	T	I	

Continuation

791	798	816	822	825	831	850/852	855	858	862
T	T	T	A	T	A		C	A	C
A	C	C	C	C	C	G C G	G	T	T
264	266	272	274	275	277	284	285	286	288
F	N	G	P	V	E	_	S	S	P
Y	.	.	.	.	D	A	.	.	S

HPV	Ramachandran Favoured	Sysbio (Mean)	GalaxyRefine
HPV 6	-	-	-
HPV 30	95.19%	-	-
HPV 35	84.26%	95.11%	-
HPV 51	84.06%	84.86%	95.14%
HPV 52	-	-	-
HPV 54	92.96%	-	-
HPV 56	94.77%	-	-
HPV 61	88.87%	91.29%	-
HPV 73	94.01%	-	-
HPV 84	90.22%	-	-
HPV 86	88.47%	87.31%	91.53%
HPV 87	87.85%	88.05%	91.47%
HPV 89	89.20%	91.20%	
HPV 114	-	-	-

HPV 35 - Sysbio	Ramachandran Favoured
Refined model 1	93.95%
Refined model 2	94.92%
Refined model 3	95.40%
Refined model 4	95.64%
Refined model 5	95.64%

HPV 51 - Sysbio	Ramachandran Favoured
Refined model 1	84.86%
Refined model 2	83.86%
Refined model 3	83.86%
Refined model 4	84.06%
Refined model 5	83.07%

HPV 87 - Galaxy Refine	Ramachandran Favoured
Refined model 1	91.43%
Refined model 2	91.43%
Refined model 3	91.43%
Refined model 4	91.24%
Refined model 5	91.83%

HPV 51 - Sysbio	Ramachandran Favoured
Refined model 1	93.38%
Refined model 2	93.82%
Refined model 3	94.04%
Refined model 4	94.26%
Refined model 5	94.48%

HPV 51 Model 5	Ramachandran Favoured
Refined model 1	94,70%
Refined model 2	94,70%

Refined model 3	95,14%
Refined model 4	95,14%
Refined model 5	95,14%

HPV 61 - Sysbio	Ramachandran Favoured
Refined model 1	90.85%
Refined model 2	91.45%
Refined model 3	91.45%
Refined model 4	91.25%
Refined model 5	91.45%

HPV 86 - Galaxy Refine	Ramachandran Favoured
Refined model 1	91.45%
Refined model 2	91.85%
Refined model 3	91.45%
Refined model 4	91.05%
Refined model 5	91.85%

HPV 89 - Sysbio	Ramachandran Favoured
Refined model 1	91.20%
Refined model 2	91.00%
Refined model 3	91.20%
Refined model 4	91.20%
Refined model 5	91.40%
